# Diffuse Pericoronary Soft-Tissue Cuffing on Coronary Computed Tomography Angiography in a Patient with Unstable Angina: Possible IgG4-Related Coronary Periarteritis

**DOI:** 10.3390/diagnostics16172683

**Published:** 2026-08-22

**Authors:** Shuo Liang, Dan Li, Hong Zhang

**Affiliations:** 1 Department of Radiology, Chest Hospital, Tianjin University, Tianjin 300222, China; movingspirit@163.com; 2 Clinical School of Thoracic, Tianjin Medical University, Tianjin 300070, China; 18348908211@163.com

**Keywords:** IgG4-related disease, coronary periarteritis, coronary computed tomography angiography, CT-derived fractional flow reserve, pericoronary soft tissue, unstable angina

## Abstract

A 66-year-old man with hypertension, type 2 diabetes mellitus, and a 50-year smoking history was presented with acute chest pain clinically consistent with unstable angina. Coronary computed tomography angiography (CCTA) showed multivessel atherosclerosis and, more strikingly, diffuse sheath-like pericoronary soft-tissue cuffing around the major epicardial arteries—an appearance reported as the “mistletoe sign” and compatible with immunoglobulin G4 (IgG4)-related coronary periarteritis. CT-derived fractional flow reserve (CT-FFR) measured 0.75 in the left anterior descending artery, 0.68 in the left circumflex artery, and 0.94 in the right coronary artery. Invasive angiography identified a 90% proximal left circumflex stenosis as the flow-limiting lesion; drug-eluting stent implantation restored Thrombolysis in Myocardial Infarction (TIMI) grade 3 flow. Serum IgG4 (145 mg/dL) was only marginally above the diagnostic threshold, and troponin was unavailable, so the diagnosis remained clinical. Without histopathology, the findings support possible rather than definite IgG4-related disease, and the contribution of the pericoronary process to the stenosis could not be established. The patient remained stable on conventional medical therapy. CCTA, CT-FFR, and angiography answer complementary questions; diffuse pericoronary change warrants serologic and systemic evaluation for inflammatory coronary involvement, with cautious etiologic attribution.

**Figure 1:**
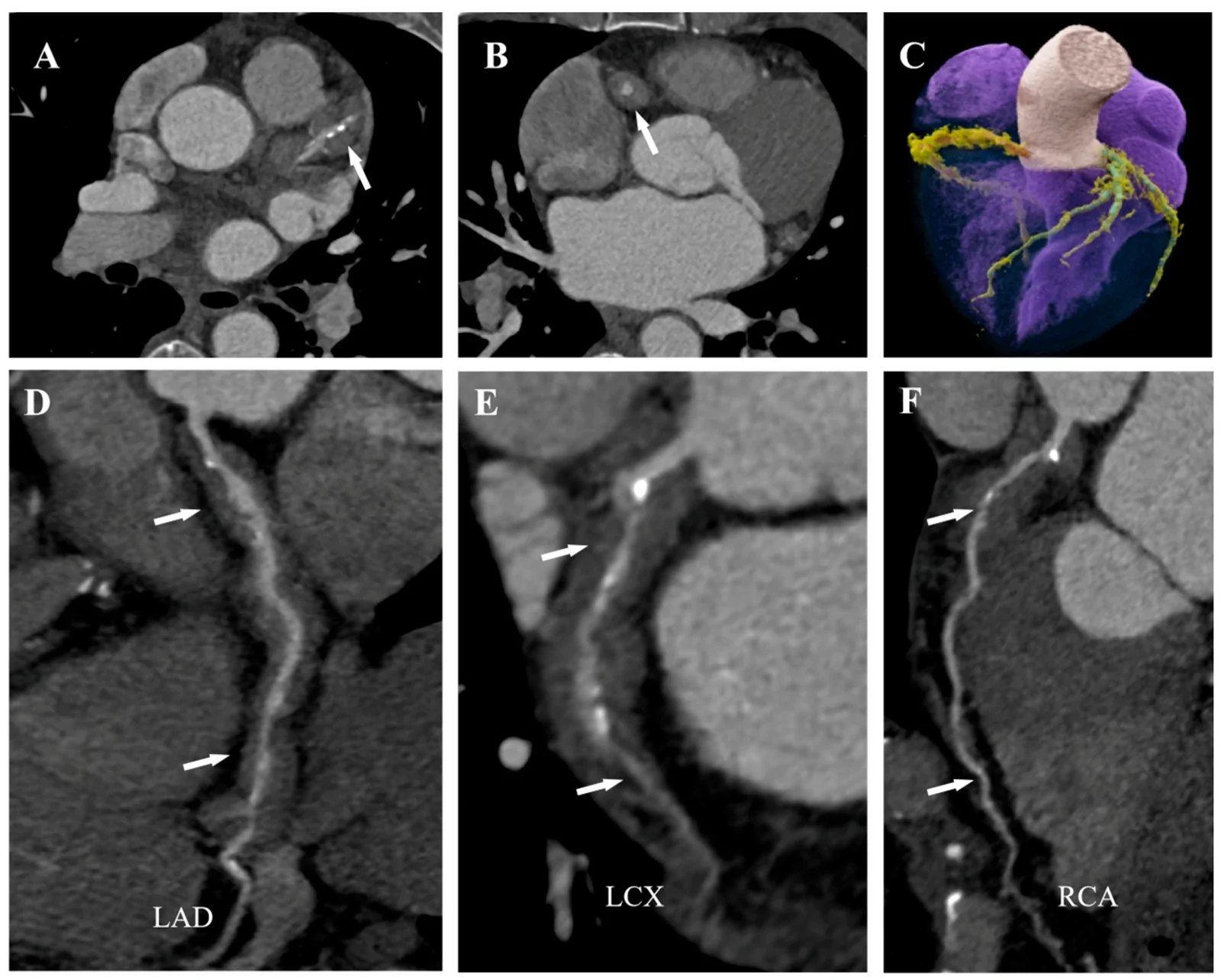
The patient reported intermittent precordial discomfort for four days, worsening over the 10 h before presentation and relieved by nitroglycerin. Blood pressure was 130/80 mmHg and heart rate 71 beats/min; the electrocardiogram showed sinus rhythm with ST-segment/T-wave changes. Cardiac troponin was not available; the working diagnosis of unstable angina was therefore clinical, and non-ST-segment elevation myocardial infarction was not excluded. Cardiovascular risk factors included hypertension for more than 10 years, type 2 diabetes mellitus for more than 3 years, a 50-year smoking history (quit 1 year earlier), and a prior gastric ulcer. No evaluation for extra-coronary organ involvement of IgG4-related disease (salivary and lacrimal glands, pancreas, biliary tree, kidneys, retroperitoneum, aorta, lymph nodes) was documented; because isolated single-organ coronary involvement is uncommon in IgG4-related disease, this absence lowers the pre-test probability of the systemic diagnosis [1]. Coronary computed tomography angiography (CCTA) with axial images (**A**,**B**), cinematic volume-rendered reconstruction (**C**), and curved planar reconstructions (**D**–**F**) in a 66-year-old man presenting with suspected unstable angina. (**A**) Axial image at the level of the left main bifurcation: calcific foci embedded in soft-tissue attenuation cuffing the proximal left coronary segments (arrow). (**B**) Axial image at the proximal right coronary artery: the contrast-filled vessel enveloped by a near-circumferential soft-tissue halo within the right atrioventricular groove fat (arrow). (**C**) Cinematic volume-rendered overview of the epicardial coronary tree; as a thresholded surface display, it omits the pericoronary soft-tissue component. (**D**–**F**) Curved planar reconstructions of the left anterior descending artery (**D**), left circumflex artery (**E**), and right coronary artery (**F**): long-segment pericoronary soft-tissue cuffing with an irregular outer margin and scattered calcifications (arrows mark the proximal and distal extent of the cuffing). Together with the elevated serum IgG4, these findings supported possible immunoglobulin G4 (IgG4)-related coronary periarteritis. Acquisition:dual-source CT scanner (SOMATOM Force; Siemens Healthineers, Forchheim, Germany) using prospective ECG-triggered acquisition. Tube voltage was selected automatically by CARE kV (range, 70–120 kV), with automated tube current modulation (CARE Dose 4D). Detector collimation was 2 × 192 × 0.6 mm and gantry rotation time was 0.25 s. Hounsfield unit measurements could not be obtained because only static exported images, without the source DICOM data, were available.

**Figure 2:**
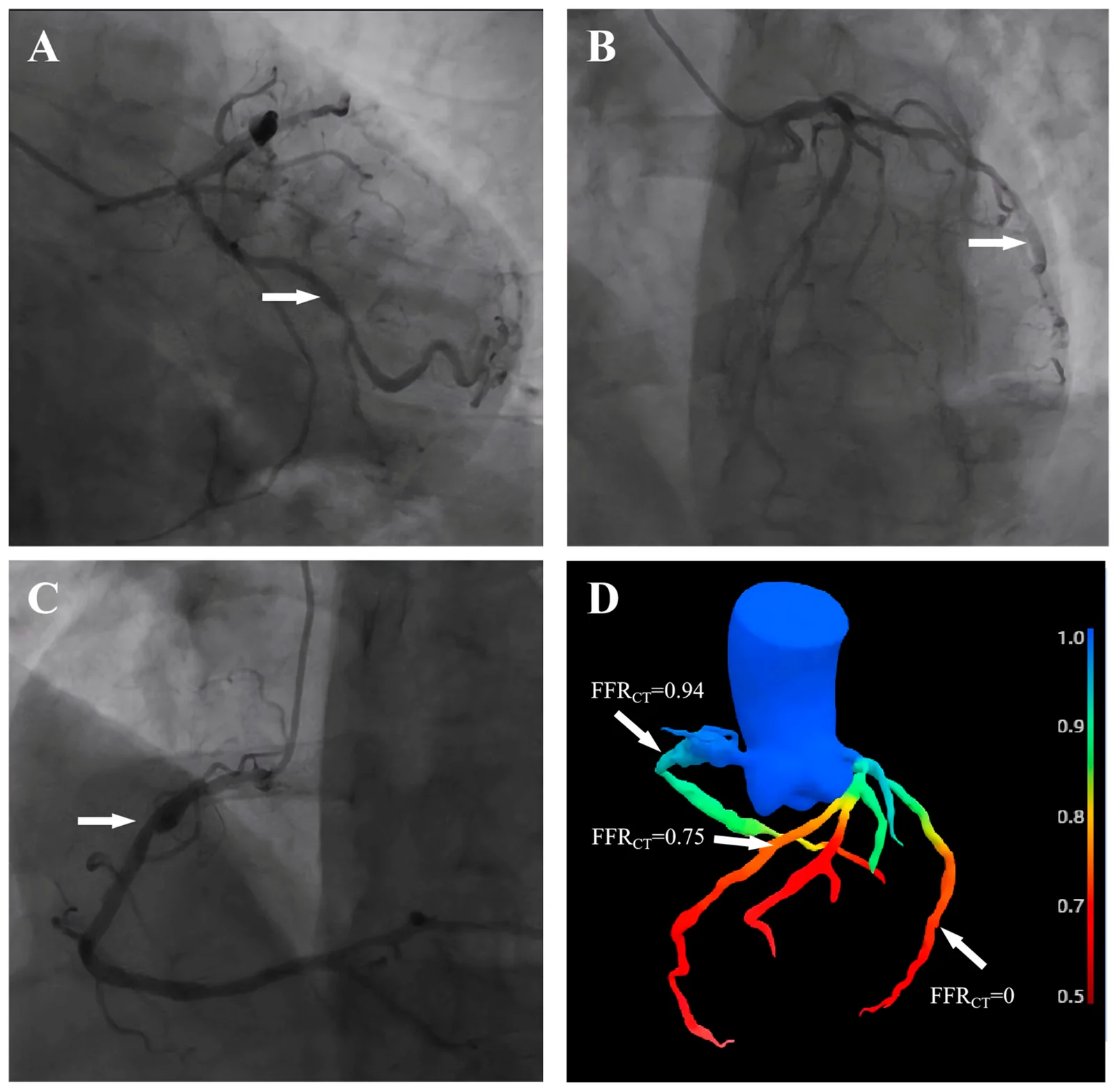
CCTA demonstrated a right-dominant coronary circulation with atherosclerotic involvement of all three epicardial territories; obstructive stenosis was confined to the proximal left circumflex artery, corresponding to CAD-RADS 2.0 category 4B [2], whereas disease in the left anterior descending and right coronary arteries was non-obstructive. More strikingly, there was diffuse soft-tissue attenuation surrounding the major epicardial coronary arteries, whose irregular outer wall contour imparted a sheath-like configuration. On the axial image at the level of the left main bifurcation (Figure 1A), calcific foci lay embedded in soft-tissue attenuation cuffing the proximal left coronary segments. The axial image at the proximal right coronary artery (Figure 1B) showed the contrast-filled vessel enveloped by a near-circumferential halo of soft tissue within the right atrioventricular groove fat. The cinematic volume-rendered overview (Figure 1C) displayed the global distribution of the epicardial coronary tree and its spatial relationship to the aortic root and cardiac silhouette; as a thresholded surface display, it omits the pericoronary soft-tissue component. Curved planar reconstructions (Figure 1D–F) delineated the longitudinal extent of the process in the left anterior descending, left circumflex, and right coronary arteries: the contrast column was surrounded over long segments by continuous soft-tissue attenuation with an irregular outer margin and scattered calcifications. The interpreting radiologist recommended clinical correlation for vasculitis or IgG4-related coronary involvement. Quantitative attenuation measurements were not obtainable: only static exported images were accessible, and the source Digital Imaging and Communications in Medicine (DICOM) files could not be retrieved, so Hounsfield unit values for the pericoronary tissue, the adjacent epicardial fat, and a reference structure could not be measured (see Figure 1 legend). Pericoronary adipose tissue attenuation (PCAT, or the fat attenuation index) is now an established CCTA marker of coronary inflammation with prognostic value [3,4] and has been used to track inflammatory activity in IgG4-related coronary periarteritis [5]; PCAT analysis was not feasible here for the same reason. Dual-energy CT has likewise been used to characterize cardiac involvement in IgG4-related disease [6]. Invasive coronary angiography revealed a 30% stenosis at the mid-segment of the left anterior descending artery ((**A**), arrow), a 90% stenosis at the proximal segment of the left circumflex artery ((**B**), arrow), and a 30% stenosis in the right coronary artery ((**C**), arrow). (**D**) CT-derived fractional flow reserve (CT-FFR) color-coded map with per-vessel annotation and color scale: 0.75 in the left anterior descending artery, 0.68 in the left circumflex artery, and 0.94 in the right coronary artery. Invasive coronary angiography, which depicts only the opacified lumen, identified a 90% proximal left circumflex stenosis as the flow-limiting lesion (stenoses graded by visual estimation); percutaneous coronary intervention with a 2.5 × 16 mm drug-eluting stent restored TIMI grade 3 flow without dissection. The diffuse pericoronary abnormality that dominated the CCTA was angiographically occult. Angiography thus defined the treatable luminal obstruction and guided intervention, whereas CCTA characterized the vessel-wall and periarterial process that luminography cannot capture. CT-FFR (Shukun CoronaryDoc-FFR, V7.7) provided a noninvasive functional readout: values were 0.75 in the left anterior descending artery and 0.68 in the left circumflex artery—at or below the conventional 0.80 threshold for hemodynamic significance—whereas the right coronary artery measured 0.94 (**D**). In a meta-analysis of 43 studies and 7291 vessels, CT-FFR achieved a pooled diagnostic accuracy of 82.2% against invasive FFR at the ≤0.80 threshold, supporting its role as an adjunct rather than a stand-alone reference standard [7]. Several explanations are plausible for a CT-FFR of 0.75 in the left anterior descending artery despite only a 30% angiographic stenosis: diffuse non-obstructive atherosclerosis with serial pressure loss, a functional contribution of the pericoronary process itself, and degraded lumen segmentation adjacent to abundant perivascular soft tissue—a recognized source of CT-FFR error [8]. Invasive FFR was not performed; it cannot be determined whether the low left anterior descending value reflects the pericoronary process or diffuse atherosclerosis, and anatomical–functional concordance is claimed only for the severe proximal left circumflex lesion. Descriptive signs for this appearance—including the “mistletoe sign”, first reported in the coronary manifestation of Ormond disease (idiopathic retroperitoneal fibrosis) [9] and only later applied to IgG4-related disease, with similar appearances described in other vasculitides [10]—are used here only as secondary descriptors, as the sign is neither standardized nor specific to IgG4-related disease. Under the 2020 revised comprehensive diagnostic criteria, definite IgG4-related disease requires characteristic clinical and radiologic features plus both serologic and histopathologic criteria, whereas characteristic features with serum IgG4 elevation alone support possible disease [11]. This patient’s serum IgG4 was 145 mg/dL (1450 mg/L), approximately 7% above the 135 mg/dL (1350 mg/L) threshold. An elevation of this marginal size is borderline and nonspecific: values in this range occur in atherosclerosis, infection, malignancy, and other vasculitides, and serum IgG4 remains an imperfect stand-alone marker with limited sensitivity and specificity [12,13]. Because this elevation carries much of the diagnostic weight here, its marginal magnitude should be read as a caution rather than as corroboration. Combined with the characteristic coronary imaging, the case is placed at the possible rather than definite level [11]; histopathology and systemic confirmatory data are unavailable, so the case remains possible rather than established IgG4-related coronary periarteritis. Wang et al. synthesized reported cases of coronary involvement in IgG4-related disease (*n* = 134), in which arterial wall thickening occurred in 43%, periarterial soft-tissue encasement in 46%, aneurysmal or ectatic change in 49%, and secondary stenosis in 47% of patients, with phenotypes frequently coexisting [14]. The present case combined the encasement and stenosis phenotypes. The left anterior descending artery was the most frequently affected vessel (79%) in that synthesis [14], although an earlier systematic review found no clear branch predilection [15], and a smaller 13-patient series reported a higher stenosis frequency (69%) [16]. These figures derive largely from case reports and retrospective series and remain susceptible to selection bias, a caveat that also applies to the 5% of the pooled cohort who died from secondary myocardial ischemia or infarction [14]. Focal atherosclerosis was identified as the direct cause of stenosis in approximately one-fifth of cases [14]. Our patient carried multiple conventional risk factors, had visible coronary calcification, and showed CT-FFR-positive lesions in two territories; whether the 90% left circumflex stenosis arose from the pericoronary process, from ordinary atherosclerosis, or from their combination could not be established without intravascular imaging or histology. Calcification does not exclude inflammatory periarteritis—calcification and stenosis each occurred in nine of 13 patients in one coronary series [16]—but pericoronary cuffing does not prove it either. Distinguishing IgG4-related coronary periarteritis from other causes of pericoronary soft-tissue encasement generally requires inflammatory markers and autoimmune serology, whole-body imaging for extra-coronary involvement, and tissue sampling where feasible [10]. The differential diagnosis of diffuse pericoronary cuffing includes other large- and medium-vessel vasculitides—notably Takayasu arteritis, giant cell arteritis, and ANCA-associated vasculitis with coronary involvement [10,17]; Erdheim–Chester disease with periarterial infiltration [18]; epicardial lymphoma or metastatic soft-tissue encasement, which typically envelops coronary arteries without stenosing them [19]; organized thrombus within an aneurysmal segment, where the soft-tissue ring lies inside the aneurysmal wall rather than around the vessel [20]; and post-inflammatory fibrosis of a healed vasculitis [10]. Circulating inflammatory mediators such as matrix metalloproteinase-8, implicated in vascular inflammation and atherosclerotic plaque destabilization [21], were not measured in this patient. In reported IgG4-related coronary involvement, 18F-fluorodeoxyglucose positron emission tomography/computed tomography (PET/CT) and cardiovascular magnetic resonance (CMR) have been used to gauge inflammatory activity and treatment response, although spatial resolution limits assessment of medium-sized coronary branches [14]; multimodality imaging of a coronary IgG4-related lesion has been described as “tumor-like” [22]. Neither PET/CT nor CMR was performed here, and a systematic vasculitis workup was not documented, so these alternatives remain untested rather than excluded. Management focused on the culprit lesion. Angiography-guided stenting treated the flow-limiting circumflex lesion; conventional medical therapy was continued thereafter. No corticosteroid or other immunosuppressive therapy was initiated for three reasons: the diagnosis remains at the possible level without histopathologic or systemic corroboration; no extra-coronary organ involvement was documented; and the flow-limiting lesion had been effectively treated, with an uncomplicated post-interventional course. A formal rheumatology or immunology consultation was not documented; multidisciplinary assessment would be the appropriate pathway should systemic corroboration emerge. It should also be noted that implanting a drug-eluting stent into a segment that may be surrounded by active inflammation carries its own risks—stent malapposition, positive remodeling, late aneurysm formation, and in-stent restenosis [23,24]—a consideration that connects directly to the unresolved sequencing question below. The patient remained clinically stable at 6 months; however, no further CCTA examination was performed. In the literature, glucocorticoids are the most commonly reported anti-inflammatory therapy, with rituximab or other immunosuppressive agents and surgical intervention reserved for selected phenotypes; the evidence base is almost entirely observational, the optimal sequencing of revascularization and anti-inflammatory therapy remains unresolved, and dedicated post-treatment coronary imaging is inconsistently performed [14,25]. Any decision to initiate immunosuppression in a patient like ours would require multidisciplinary assessment and, where feasible, histopathologic or systemic corroboration. IgG4-related disease could not be established, and the contribution of the pericoronary process to the flow-limiting stenosis remains undetermined. Definitive classification requires histopathologic and systemic corroboration. In summary, CCTA, CT-FFR, and invasive angiography answer complementary questions: CCTA characterizes the vessel-wall and periarterial process, CT-FFR provides a noninvasive functional readout, and angiography defines and guides treatment of the flow-limiting obstruction. Diffuse pericoronary soft-tissue change in acute coronary syndromes warrants serologic and systemic evaluation for inflammatory coronary involvement; in patients with conventional risk factors, etiologic attribution should remain cautious, and descriptive signs such as the “mistletoe sign” should be treated as secondary, nonspecific descriptors.

## Data Availability

The original contributions presented in this study are included in the article. Further inquiries can be directed to the corresponding author.

## Abbreviations

ANA	Antinuclear antibody
ANCA	Antineutrophil cytoplasmic antibody
CAD-RADS	Coronary Artery Disease–Reporting and Data System
CCTA	Coronary computed tomography angiography
CMR	Cardiovascular magnetic resonance
CPR	Curved planar reconstruction
CRP	C-reactive protein
CT-FFR	CT-derived fractional flow reserve
cVRT	Cinematic volume rendering technique
DICOM	Digital Imaging and Communications in Medicine
ESR	Erythrocyte sedimentation rate
FFR	Fractional flow reserve
IgG4	Immunoglobulin G4
PCAT	Pericoronary adipose tissue attenuation
PET/CT	Positron emission tomography/computed tomography
TIMI	Thrombolysis in Myocardial Infarction
